# Association of Self-Reported Sleep Metrics With Imaging Markers of Small Vessel Disease and Cognition in Patients With TIA or Mild Stroke

**DOI:** 10.1212/WNL.0000000000213734

**Published:** 2025-05-28

**Authors:** Dillys Xiaodi Liu, Mary Sau-Man Ip, David Chi-Leung Lam, Francesca M. Chappell, Una Clancy, Daniela Jaime Garcia, Carmen Arteaga-Reyes, Maria Del C. Valdés Hernández, Michael Thrippleton, Michael S. Stringer, Yajun Cheng, Junfang Zhang, Fergus Doubal, Gary Kui Kai Lau, Joanna M. Wardlaw

**Affiliations:** 1 Division of Neurology, Department of Medicine, LKS Faculty of Medicine, The University of Hong Kong, China;; 2 Division of Respiratory Medicine, Department of Medicine, LKS Faculty of Medicine, The University of Hong Kong, China;; 3 Centre for Clinical Brain Sciences, University of Edinburgh, United Kingdom;; 4 UK Dementia Research Institute, Centre at the University of Edinburgh, United Kingdom;; 5 Edinburgh Imaging Facility RIE, University of Edinburgh, United Kingdom;; 6 Department of Neurology, West China Hospital, Sichuan University, Chengdu;; 7 Department of Neurology & Institute of Neurology, Ruijin Hospital affiliated with Shanghai Jiao Tong University School of Medicine, China; and; 8 Laboratory of Neuropsychology and Human Neuroscience, The University of Hong Kong, China.

## Abstract

**Background and Objectives:**

Disturbed sleep is common after stroke, yet its relationship with cerebral small vessel disease (SVD) and cognitive performance in the stroke population, particularly patients with TIA/mild stroke who are on the milder end of the cerebrovascular spectrum, remains understudied. We aim to examine the associations of self-reported sleep metrics with neuroimaging markers of SVD and cognitive performance in patients with TIA/mild stroke from 2 prospective stroke cohorts.

**Methods:**

We studied adult patients with TIA/mild stroke (NIH Stroke Scale [NIHSS] score <7) who were consecutively recruited from Mild Stroke Study 3 (MSS3, University of Edinburgh) and the stroke cohort (the University of Hong Kong, HKU) during 2018–2022. Both MSS3 (N = 211) and HKU (N = 211) cohorts assessed SVD burden visually on brain MRI, cognitive performance using Montreal Cognitive Assessment (MoCA), and sleep quality using a structured sleep questionnaire at baseline visit. The primary outcomes were SVD markers, and the secondary outcome was total MoCA score. The associations of sleep metrics with SVD markers and cognitive performance were assessed using regression models, adjusted for demographics, vascular risk factors, history of depression and stroke, and study sites.

**Results:**

In 422 patients (65.6 ± 11.8 years, 67% male, median NIHSS score 1.0), longer in-bed time was independently associated with greater global SVD and Fazekas periventricular white matter hyperintensity (WMH) burden: odds ratio (OR)_summary SVD score_ = 1.27 per 1-SD increase (95% CI 1.05–1.53), false discovery rate (FDR)–adjusted *p* = 0.04; OR_periventricular WMH_ = 1.53 per 1-SD increase (95% CI 1.18–2.00), *p* = 0.003. Longer sleep duration was independently associated with presence of cerebral microbleeds: OR = 1.42 per 1-SD increase (95% CI 1.09–1.87), *p* = 0.04. Longer in-bed time was associated with a lower total MoCA score after covariate adjustment: standardized β = −0.58 (95% CI −0.99 to −0.16), *p* = 0.02.

**Discussion:**

Disturbed sleep, including longer in-bed time and longer sleep duration, was cross-sectionally associated with greater SVD burden and worse cognitive performance in patients with TIA/mild stroke. Future longitudinal studies are warranted to validate our findings.

## Introduction

Disturbed sleep is common among patients with stroke and often presents with prolonged time in bed, insomnia, hypersomnia, and excessive daytime sleepiness.^1^ It is estimated that up to 38% of stroke patients have insomnia^2^ and 27% have sleep needs longer than 10 hours per day.^1^ Disturbed sleep may be linked to adverse brain health through worsening vascular disease or disrupting glymphatic function, according to a Scientific Statement from the American Heart Association.^3^ Yet, its role in brain health in the stroke population, particularly patients with TIA/mild stroke who are on the milder end of the cerebrovascular spectrum, remains understudied.

Cerebral small vessel disease (SVD), characterized by abnormal small perforating arterioles and capillaries, is a major cause of stroke and vascular dementia worldwide.^4^ SVD imaging markers include recent small subcortical infarcts, white matter hyperintensities (WMHs), lacunes, enlarged perivascular spaces (PVSs), cerebral microbleeds, and brain atrophy.^5^ In middle-aged and older adults, poor sleep quality has been associated with worse WMHs,^6^ brain atrophy,^7^ and more PVSs,^8-10^ but not with cerebral microbleeds or lacunes,^6^ and longitudinally with increasing WMHs in older people.^11^ Nonetheless, these studies reported inconsistent findings or comprised stroke-free individuals with small sample sizes in single cohorts. Moreover, despite the gender difference in sleep pattern,^12^ SVD burden,^13^ and vascular cognitive impairment,^14^ research on gender differences in the relationships between sleep and brain health in stroke populations is limited.

In this study, we explored the associations of self-reported sleep metrics with a full range of SVD markers and cognitive performance in patients with TIA/mild stroke, from 2 prospective stroke cohorts reflecting different ethnic backgrounds. We hypothesize that disturbed sleep is cross-sectionally associated with greater SVD burden and worse cognitive performance across 2 stroke cohorts, with sex as a potential moderator.

## Methods

### Study Participants

We used data from 2 prospective stroke cohorts: Mild Stroke Study 3 (MSS3)^15^ (N = 211) at the University of Edinburgh and the TIA/mild stroke cohort at the University of Hong Kong (HKU) (N = 211). Both studies recruited consecutive adult patients with TIA or mild ischemic stroke (NIH Stroke Scale [NIHSS] score <7) from 2018 to 2022, diagnosed based on clinical stroke syndromes and imaging findings, from the NHS Lothian clinical stroke services, Edinburgh, United Kingdom, or the Acute Stroke Unit/TIA Clinic of Queen Mary Hospital, Hong Kong, respectively. Patients were excluded if they were diagnosed with major stroke (NIHSS score ≥7) or were not available to complete a sleep questionnaire or brain MRI. All participants had baseline assessment at 1–3 months after stroke including demographics, medical history, 3T brain MRI, sleep, and cognitive and NIHSS assessments.

### Standard Protocol Approvals, Registrations, and Patient Consents

MSS3 was approved by the South East Scotland Research Ethics Committee (reference 18/SS/0044). The HKU cohort study was approved by the Institutional Review Board of the University of Hong Kong/Hospital Authority Hong Kong West Cluster (UW18-361). All study participants gave written informed consent.

### Sleep Metric Analysis

We extracted several self-reported sleep metrics from an adapted version of the Pittsburgh Sleep Quality Index at baseline visit,^16^ a well-validated sleep questionnaire assessing sleep quality using subjective ratings among patients with stroke within a one-month time interval.^17^ Several quantitative sleep metrics including in-bed time (hours), nighttime sleep duration (hours), sleep latency (minutes), and sleep efficiency (sleep duration/in-bed time × 100%) were calculated for further analysis.

### MRI Acquisitions and Analysis

All participants underwent brain MRI at baseline. MRI sequences included 3D T1-weighted, T2-weighted, fluid-attenuated inversion recovery, susceptibility-weighted, and diffusion-weighted imaging, as described previously.^15,18^ An experienced team, supervised by a neuroradiologist (J.M.W.) and neurologist (K.K.L.), visually assessed neuroimaging markers of SVD using STRIVE criteria,^5^ including presence of lacunes, WMH burden (Fazekas scores 0–3) in deep and periventricular regions, PVSs in basal ganglia and centrum semiovale regions (BG-PVS and CSO-PVS, categorized as ≤10, 11–20, >20), and presence of cerebral microbleeds using validated visual rating scales.^19-21^ The sum of SVD score was calculated based on WMHs, PVSs, cerebral microbleeds, and lacunes as previously described.^22^ We also assessed brain atrophy in the deep (ventricular enlargement) and superficial (gyral enlargement) brain regions against a validated normal aging reference template, ranging from 1 (normal) to 6 (severe atrophy).^23^

### Cognitive Assessment

All the participants underwent the Montreal Cognitive Assessment (MoCA)^24^ at baseline, a validated screening tool for vascular cognitive impairment after acute stroke,^25^ to evaluate global cognition.

### Covariates

The following covariates were preselected, based on known confounders of sleep and SVD burden or cognition in literature review: age (years), sex (male and female), educational level (categorized as 0–6, 7–9, 10–12, and >12 years), vascular risk score (a composite sum score comprising presence of hypertension, hyperlipidemia, diabetes, and ever-smoking), history of depression, history of stroke, and study site (MSS3 or HKU cohort).

### Statistical Analysis

We described the study population using mean and SDs or median (interquartile range [IQR]) for continuous variables, or relative frequencies for categorical variables, respectively. We compared the difference in demographic, sleep, and imaging characteristics between 2 stroke cohorts using the Student *t* test, Wilcoxon rank-sum test, χ^2^ test, or Fisher exact test, as appropriate. We also performed correlation analysis to explore the linear relationship between sleep duration and other sleep metrics.

To examine the associations between sleep metrics and SVD burden, we used multivariable ordinal (summary SVD score [0–4], WMH [0, 1–2, 3] or PVS [≥10, 11–20, >20] burden, tertiles of the total brain atrophy score [sum of brain atrophy score in deep and superficial regions: 0–4, 5–8, 9–12]) or binomial (presence of lacunes or cerebral microbleeds) logistic regression analysis, with each SVD marker as the dependent variable and each sleep metric as the independent variable. We also used a linear regression model to examine the associations between each sleep metric and total MoCA score, respectively. All the sleep metrics and MoCA score were converted into *z*-scores. We applied 2 multivariable models: model 1 corrected for age, sex, and study site; model 2 further corrected for vascular risk score, education, history of depression, and history of stroke. We further examined whether sex moderates the associations between sleep and SVD burden or cognitive performance by including an interaction term for sleep metric × sex, respectively.

All statistical analyses were performed using R (version 4.2.2, R Foundation for Statistical Computing, Vienna, Austria) and Python (version 3.11.3, Python Software Foundation, Wilmington, Delaware). In the regression models, we reported odds ratios (ORs) or standardized beta coefficients with its 95% CIs and *p* values. We computed a false discovery rate (FDR) to account for multiple comparisons and used *q* = 0.05 as the significance threshold.

### Data Availability

The anonymized data that support the findings of this study are available from the corresponding authors on reasonable request.

## Results

### Study Sample Characteristics

**Figure 1:**
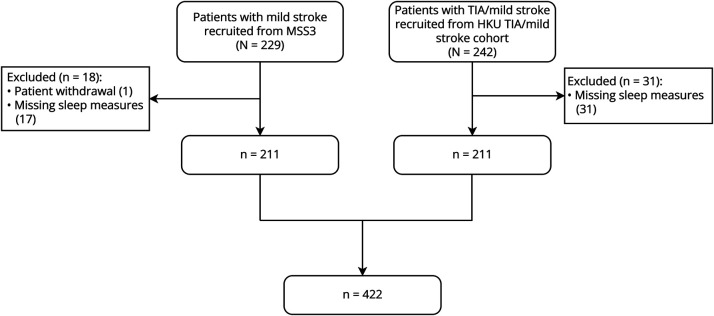
Study Flowchart HKU = University of Hong Kong; MSS3 = Mild Stroke Study 3.

There were 422 participants included in the final analysis (Figure 1). The study sample had a mean age of 65.4 ± 11.8 years, 66% were male, and the median NIHSS score was 1.0 (IQR 2.0). The MSS3 cohort was predominantly White British while the HKU cohort was predominantly Chinese. Compared with participants in the HKU cohort, MSS3 participants had similar mean age and were more likely to have higher educational level, history of depression, smoking history, hyperlipidemia, presence of lacunes, higher deep WMH and PVS burden, and worse brain atrophy, but less likely to have diabetes (all *p* < 0.05, Table 1). In addition, MSS3 participants had longer total in-bed time, longer sleep duration, and lower sleep efficiency (all *p* < 0.05, Table 1).

**Table 1:** Baseline Demographic, Clinical, and Imaging Characteristics of the 2 Stroke Cohorts

Characteristic	Overall (N = 422)	HKU TIA/mild stroke cohort, Hong Kong (N = 211)	MSS3, Edinburgh, United Kingdom (N = 211)	*p* Value
Demographics				
Age, y	65.4 (11.8)	64.9 (12.4)	65.9 (11.2)	0.43
Male	278 (66)	140 (66)	138 (65)	0.92
Education years				<0.001
0–6 y	68 (16)	68 (32)	0 (0)	
7–9 y	94 (22)	40 (19)	54 (26)	
10–12 y	134 (32)	63 (30)	71 (34)	
>12 y	126 (30)	40 (19)	86 (41)	
Clinical				
NIHSS score	1.0 (2.0)	1.0 (2.0)	1.0 (2.0)	0.42
Hypertension	109 (26)	64 (30)	45 (21)	0.045
Hyperlipidemia	267 (63)	124 (59)	143 (68)	0.07
Diabetes	230 (55)	74 (35)	156 (74)	<0.001
Previous TIA/stroke	52 (12)	19 (9.0)	33 (16)	0.05
Atrial fibrillation	38 (9.0)	19 (9.0)	19 (9.0)	1
Depression	50 (12)	3 (1.4)	47 (22)	<0.001
Ever-smoker	185 (44)	65 (31)	120 (57)	<0.001
Alcohol drinker	104 (25)	45 (21)	59 (28)	0.14
MoCA score	23.7 (5.0)	22.9 (6.1)	24.5 (3.6)	0.18
Imaging				
Summary SVD score				0.18
0	118 (28)	65 (31)	53 (25)	
1	99 (23)	55 (26)	44 (21)	
2	94 (22)	38 (18)	56 (27)	
3	69 (16)	34 (16)	35 (17)	
4	42 (10)	19 (9.0)	23 (11)	
Presence of lacunes	183 (43)	80 (38)	103 (49)	0.024
Presence of microbleeds	75 (18)	37 (18)	38 (18)	0.90
Deep WMH				0.05
Fazekas score 0	50 (12)	32 (15)	18 (8.5)	
Fazekas scores 1–2	316 (75)	148 (70)	168 (80)	
Fazekas score 3	56 (13)	31 (15)	25 (12)	
Periventricular WMHs				0.15
Fazekas score 0	10 (2.4)	2 (0.9)	8 (3.8)	
Fazekas scores 1–2	319 (76)	163 (77)	156 (74)	
Fazekas score 3	93 (22)	46 (22)	47 (22)	
Basal ganglia PVSs				<0.001
≤10	182 (43)	102 (48)	80 (38)	
11–20	165 (39)	88 (42)	77 (36)	
>20	75 (18)	21 (10)	54 (26)	
Central semiovale PVSs				<0.001
≤10	174 (41)	94 (45)	80 (38)	
11–20	132 (31)	86 (41)	46 (22)	
>20	116 (27)	31 (15)	85 (40)	
Tertile of total brain atrophy score				0.019
0–4	179 (42)	102 (48)	77 (36)	
5–8	165 (39)	69 (33)	96 (45)	
9–12	78 (18)	40 (19)	38 (18)	
Sleep				
Total in-bed time, h	8.5 (1.7)	8.0 (1.6)	9.1 (1.6)	<0.001
Sleep duration, h	6.8 (1.5)	6.7 (1.4)	7.0 (1.6)	0.04
Sleep latency, min	26.0 (29.9)	26.9 (35.1)	25.2 (23.5)	0.11
Sleep efficiency, %	81.4 (15.8)	85.4 (15.2)	77.5 (15.5)	<0.001

Abbreviations: IQR = interquartile range; MoCA = Montreal Cognitive Assessment; NIHSS = NIH Stroke Scale; PVSs = perivascular spaces; SVD = small vessel disease; WMH = white matter hyperintensity.

Data are presented as mean (SD), median (IQR), or n (%).

### Correlation of Sleep Metrics

Both in-bed time and sleep efficiency were positively correlated with sleep duration: in-bed time: *r* = 0.52, *p* < 0.001; sleep efficiency: *r* = 0.56, *p* < 0.001. Sleep latency was negatively correlated with sleep duration (*r* = −0.24, *p* < 0.001).

### Sleep and SVD Markers

**Figure 2:**
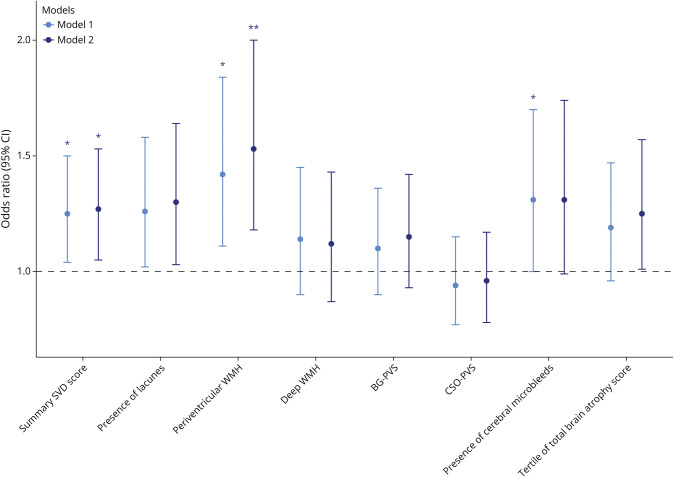
Adjusted Odds Ratios With 95% CI for In-Bed Time and SVD Markers Model 1 adjusted for age, sex, and study site. Model 2 further adjusted for vascular risk score, education, history of depression, and history of stroke. Data are presented as odds ratio (95% CI) with FDR-adjusted *p* value. *FDR-adjusted *p* < 0.05; ** FDR-adjusted *p* < 0.01. BG = basal ganglia; CSO = central semiovale; FDR = false discovery rate; PVS = perivascular space; SVD = small vessel disease; WMH = white matter hyperintensity.

In the pooled analysis, in-bed time was associated with all SVD markers except for deep WMHs and presence of cerebral microbleeds, when adjusting for age, sex, and study site (Figure 2, model 1). The associations remained significant after full adjustment for in-bed time with summary SVD score and periventricular WMHs (OR_summary SVD score_ = 1.27 per 1-SD increase [95% CI 1.05–1.53], FDR-adjusted *p* = 0.04; OR_periventricular WMHs_ = 1.53 per 1-SD increase [95% CI 1.18–2.00], *p* = 0.003) while did not reach statistical significance with presence of lacunes or brain atrophy (OR_presence of lacunes_ = 1.30 per 1-SD increase [95% CI 1.03–1.64], *p* = 0.06; OR_tertile of total brain atrophy score_ = 1.25 per 1-SD increase [95% CI 1.01–1.57], *p* = 0.07; Figure 2, model 2).

**Figure 3:**
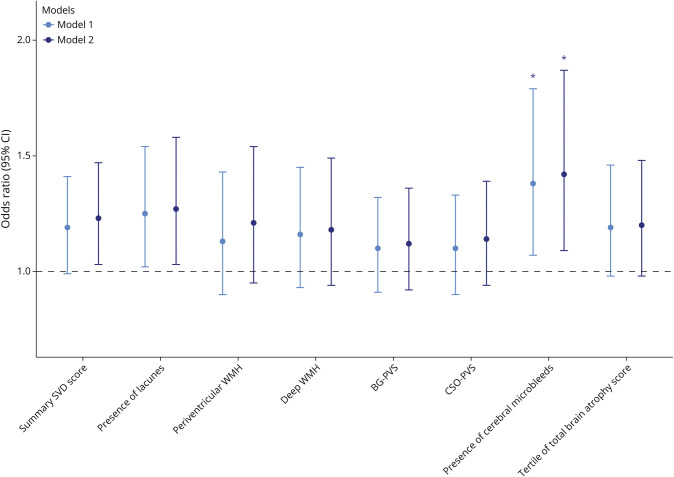
Adjusted Odds Ratios With 95% CI for Sleep Duration and SVD Markers Model 1 adjusted for age, sex, and study site. Model 2 further adjusted for vascular risk score, education, history of depression, and history of stroke. Data are presented as odds ratio (95% CI) with FDR-adjusted *p* value. *FDR-adjusted *p* < 0.05. BG = basal ganglia; CSO = central semiovale; FDR = false discovery rate; PVS = perivascular space; SVD = small vessel disease; WMH = white matter hyperintensity.

Sleep duration was associated with presence of lacunes and cerebral microbleeds in model 1 (Figure 3, model 1). The association only remained significant for presence of cerebral microbleeds after full adjustment (OR_presence of cerebral microbleeds_ = 1.42 per 1-SD increase [95% CI 1.09–1.87], *p* = 0.04; OR_presence of lacunes_ = 1.27 per 1-SD increase [95% CI 1.03–1.58], *p* = 0.07; Figure 3, model 2). Similar to the patterns observed for in-bed time, a positive association was found between sleep duration and both summary SVD score and brain atrophy, although these associations did not reach statistical significance. No significant association was found between sleep duration and other SVD markers (Figure 3, model 2).

In addition, we did not find any association between sleep latency or sleep efficiency and SVD markers, respectively (eTable 1).

### Sleep and Cognitive Performance

Pooled estimates revealed that longer in-bed time, rather than longer sleep duration, was associated with a lower total MoCA score after covariate adjustment (standardized β_total time in bed_ = −0.58 [95% CI −0.99 to −0.16], *p* = 0.02; standardized β_sleep duration_ = −0.22 [95% CI −0.61 to 0.17], *p* = 0.48; Table 2, model 2). No significant associations were detected between sleep latency or sleep efficiency and total MoCA score (all *p* > 0.05) (Table 2, model 2).

**Table 2:** Association Between Sleep Metrics and Total MoCA Score

Sleep metrics (z-score)	Model 1	Model 2
In-bed time, h	−0.15 (−0.24 to −0.05)**	−0.58 (−0.99 to −0.16)*
Sleep duration, h	−0.02 (−0.11 to 0.07)	−0.22 (−0.61 to 0.17)
Sleep efficiency, %	0.12 (0.03 to 0.21)*	0.32 (−0.09 to 0.73)
Sleep latency, min	−0.12 (−0.21 to −0.04)*	−0.44 (−0.82 to −0.05)

Abbreviations: FDR = false discovery rate; MoCA = Montreal Cognitive Assessment.

Data are presented as standardized β (95% CI) with FDR-adjusted *p* value. Model 1 adjusted for age, sex, and study site; model 2 further adjusted for vascular risk score, education, history of depression, and history of stroke.

*FDR-adjusted *p* < 0.05; ** FDR-adjusted *p* < 0.01.

### Moderation Analysis

There was no moderating effect of sex on the associations between self-reported sleep metrics and SVD burden (eTable 2) or total MoCA score (eTable 3).

## Discussion

In this pooled analysis of 2 prospective stroke cohorts, we examined cross-sectional associations between self-reported sleep metrics, SVD markers, and cognitive performance at 1–3 months after TIA/mild stroke. Our results indicated that longer in-bed time was associated with greater global SVD and periventricular WMH burden, and worse cognitive performance. Longer sleep duration was associated with presence of cerebral microbleeds, but not with cognitive performance.

Increasing evidence has linked poor sleep quality to SVD burden, mainly WMH load in community-dwelling populations,^10,11,26-29^ as well as PVS burden^9,11^ and brain atrophy^7^ in older adults. In the Northern Manhattan Study, long sleep (≥9 hours) was independently associated with a greater WMH volume.^26^ In the Atahualpa Project, poor sleep was associated with severity of WMHs, but not with lacunes or cerebral microbleeds.^6^ Furthermore, a U-shape relationship between sleep duration and WMHs was recently revealed in the UK Biobank analysis.^29,30^ While short sleep duration has been thought of as potentially leading to or a marker of adverse brain health, other markers of impaired sleep quality may be more sensitive to SVD burden in patients suffering from cerebrovascular disease. Our findings suggest that longer in-bed time is associated with greater global SVD and periventricular WMH burden in patients with TIA or mild stroke. Similar trends were observed for associations of both in-bed time and nighttime sleep duration with presence of lacunes and brain atrophy, but these did not reach statistical significance. In addition, longer sleep duration was associated with an increased risk of cerebral microbleeds. Discrepancies may stem from differences in demographics (age, sex, ethnicity), sleep patterns, vascular risk factors, and SVD burden between community populations and stroke patients because community-dwelling individuals are generally healthier. Future prospective studies are needed to explore the longitudinal effects of prolonged sleep on brain health in community-dwelling vs stroke population.

Poor sleep quality, as measured with actigraphy or a sleep questionnaire, was cross-sectionally and longitudinally associated with cognitive impairment after stroke.^31,32^ Consistent with these findings, our results indicate that longer in-bed time is associated with worse cognitive performance after stroke. Future large longitudinal studies are needed to confirm whether a consistent pattern exists between longer sleep pattern and worse cognitive performance after stroke.

The mechanism linking prolonged sleep patterns to brain health is not clear. Disturbed sleep may disrupt waste clearance including β-amyloid clearance, accentuating endothelial blood-brain barrier dysfunction and neuroinflammation,^33,34^ and eventually lead to SVD development and cognitive impairment. Excessive sleep patterns may be a marker of circadian dysfunction, which has recently been shown to affect blood pressure regulation and the onset or progression of hypertension.^35^ This, in turn, could ultimately contribute to SVD burden.^36^ In addition, by disrupting brain connections or through strategically placed lesions, SVD lesions themselves could contribute to sleep impairment and potentially worsen brain health. Moreover, white matter changes are associated with apathy and gait decline,^37,38^ which may similarly contribute to prolonged in-bed time. Longer in-bed time, in turn, could be a symptom of greater SVD burden. Future studies are needed to clarify the specific pathophysiologic processes linking longer sleep pattern to distinct SVD pathologies and vice versa.

Gender difference has been reported in sleep patterns, SVD burden, and vascular cognitive impairment. Women tend to have better polysomnography-defined sleep than men.^12^ Moreover, women more often had pronounced WMH burden while men more often had presence of lacunes among patients with vascular cognitive impairment.^14^ Only 1 study reported that longer sleep latency was associated with poor cognitive performance in men, but not in women.^39^ Although we did not find any significant moderating effect of sex, our results should be validated in future longitudinal studies.

The strengths of our study include the pooled analysis of Asian and European stroke populations, comprehensive assessment of SVD markers, and availability of information on potential confounders. Limitations include the cross-sectional design and the use of visual assessments rather than computational measures to describe WMH burden and brain atrophy. In addition, we did not exclude patients with a history of sleep apnea or track potential changes in sleep quality before and after stroke because of a lack of clinical data. Our results should be interpreted with caution because we only examined baseline cross-sectional data, which cannot establish causal relationships. Some relationships between sleep, SVD, and cognition may differ in a longitudinal context and at different time points after stroke.

In conclusion, 2 markers of disturbed sleep, longer in-bed time and longer sleep duration, were cross-sectionally associated with greater SVD burden and worse cognitive performance in patients with TIA/mild stroke.

## Glossary

BG: basal ganglia
CSO: central semiovale
FDR: false discovery rate
HKU: University of Hong Kong
IQR: interquartile range
MoCA: Montreal Cognitive Assessment
MSS3: Mild Stroke Study 3
OR: odds ratio
PVS: perivascular space
SVD: small vessel disease
WMH: white matter hyperintensity
